# Dressing wear time after breast reconstruction: study protocol for a randomized controlled trial

**DOI:** 10.1186/1745-6215-14-58

**Published:** 2013-02-22

**Authors:** Daniela Francescato Veiga, Joel Veiga-Filho, Carlos Américo Veiga Damasceno, Edilaine Maria Leci Sales, Thiago Bezerra Morais, Wânia Eliza Almeida, Neil Ferreira Novo, Lydia Masako Ferreira

**Affiliations:** 1 Division of Plastic Surgery, Department of Surgery, Universidade Federal de São Paulo, São Paulo, Brazil; 2 Division of Plastic Surgery, Department of Surgery, Universidade do Vale do Sapucaí, Pouso Alegre, Brazil; 3 Department of Microbiology, Universidade do Vale do Sapucaí, Pouso Alegre, Brazil; 4 Medical School, Universidade do Vale do Sapucaí, Pouso Alegre, Brazil; 5 Department of Bioestatistics, Universidade do Vale do Sapucaí, Pouso Alegre, Brazil; 6 Division of Plastic Surgery, Universidade Federal de São Paulo, Rua Napoleão de Barros, 715, 4º andar, CEP 04024-002, São Paulo, SP, Brazil

**Keywords:** Breast cancer, Breast reconstruction, Postoperative care, Dressings, Surgical wound infection, Bacterial growth, Patient satisfaction

## Abstract

**Background:**

One of the major risk variables for surgical site infection is wound management. Understanding infection risk factors for breast operations is essential in order to develop infection-prevention strategies and improve surgical outcomes. The aim of this trial is to assess the influence of dressing wear time on surgical site infection rates and skin colonization. Patients’ perception at self-assessment will also be analyzed.

**Methods/Design:**

This is a two-arm randomized controlled trial. Two hundred breast cancer patients undergoing immediate or delayed breast reconstruction will be prospectively enrolled. Patients will be randomly allocated to group I (dressing removed on postoperative day one) or group II (dressing removed on postoperative day six). Surgical site infections will be defined by standard criteria from the Centers for Disease Control and Prevention (CDC). Skin colonization will be assessed by culture of samples collected at predefined time points. Patients will score dressing wear time with regard to safety, comfort and convenience.

**Discussion:**

The evidence to support dressing standards for breast surgery wounds is empiric and scarce. CDC recommends protecting, with a sterile dressing for 24 to 48 hours postoperatively, a primarily closed incision, but there is no recommendation to cover this kind of incision beyond 48 hours, or on the appropriate time to shower or bathe with an uncovered incision. The results of the ongoing trial may support standard recommendations regarding dressing wear time after breast reconstruction.

**Trial registration:**

ClinicalTrials.gov identifier: http://NCT01148823.

## Background

Surgical site infection (SSI) is a relevant problem in surgical practice, with surrounding issues that still need to be clarified [1-4]. Interventions to reduce the incidence of SSI are essential to reduce not only morbidity, but also costs to the individual and to society [2,5-7].

The infection risk for clean wounds is estimated to be 1 to 2% [8]. However, SSI rates following breast surgery seem to be much higher than that expected for clean surgical procedures and range from 1 to 30% in the literature [7,9-11]. Furthermore, SSI rates after breast cancer surgical procedures are more common than SSI after non-cancer-related breast operations [7]. The incidence in the literature of SSI after mastectomy varies from 1% to 28% [5,12-14], and SSI rates after breast reconstructive procedures range from 6.3% to 28% [5,7,15-18].

Identifying SSI risk factors for breast surgery is essential in order to develop infection-prevention strategies and improve surgical outcomes [7]. Risk factors for SSI are usually classified into preoperative (patient-related, for example, age, obesity, tobacco use, comorbidities, use of immunosuppressive medications), perioperative (procedure-related factors, such as type and duration of the operation, hypoxia, operating room traffic and operating room parameters) and postoperative categories [2,19]. One of the major risk factors in the postoperative period is wound management [2,19-21]. Despite the importance of surgical wound management in preventing infection, literature on incisional wound management is sparse [21,22].

Postoperative wound care is an ancient practice, with recorded evidence dating back 4,000 years [23]. Purposes for wound dressing include protection of the wound from trauma and contamination, absorption of wound exudates and compression to minimize edema and obliterate dead space [23-27].

The search for an ideal postsurgical breast dressing has led to the development of several different materials and application techniques [28,29]. Despite the abundance of wound dressing products available nowadays, there is little empiric evidence to guide product choice for site-specific incisional wounds [26,30], and traditional low-technology gauze-based dressings are commonly used [25,27,29,31,32]. Due to lower costs, this kind of dressing is largely used in the public health system in Brazil.

From the literature, the ideal dressing wear time is also controversial. Some authors recommend the early exposure of the surgical wound, to allow easy wound inspection without inconvenience to the patient, to release the patient for his/her routine personal care and to decrease costs [21,22,24]. Other authors recommend the use of dressings for a longer time, frequently until sutures are removed, without increase in SSI rates [6,31,33,34].

The Centers for Disease Control and Prevention (CDC) provide recommendations concerning prevention of SSIs. No recommendation is offered for some practices, either because there is a lack of consensus regarding their efficacy or because the available scientific evidence is insufficient to support their adoption [19].

Available evidence to support dressing standards for incisional wounds, including breast surgery wounds, is empiric and scarce [20,23,24,26,27,30,35]. CDC’s guidelines instruct that wounds that are closed primarily should be covered with a sterile dressing for 24 to 48 hours [19]. There is neither recommendation of the type of dressing nor the ideal dressing wear time following breast surgery. These remain unsolved issues, and decisions regarding routine dressing are made on the basis of surgeons’ personal experience [19].

This randomized controlled trial was designed to assess the influence of dressing wear time after breast reconstruction on SSI rates, skin colonization and patients’ perceptions of safety, comfort and convenience.

## Methods/Design

### Study aims

To assess whether dressing wear time after breast reconstruction will influence SSI rates. Secondary aims are to assess the influence of dressing wear time on skin colonization and patients’ perceptions with regard to safety, comfort and convenience.

### Ethical issues

The Universidade do Vale do Sapucaí Ethical Committee approved the study protocol (786/07 and 1623/11). Only participants who have agreed to provide written informed consent will be included in the study.

### Study design and setting

This is a two-arm parallel group randomized controlled trial, to be conducted in a university-affiliated hospital. Patients will be recruited from the Breast Unit of the Plastic Surgery Division of the Hospital das Clínicas Samuel Libânio - Universidade do Vale do Sapucaí. This trial was registered in ClinicalTrials.gov (NCT01148823).

### Sample size

SSI rates following breast reconstruction vary in the literature, from 1% a 30% [1,4,5,9-11]. We considered clinically relevant a 10% difference in SSI rate [35]. Specifying an acceptable Type 1 error of 5%, and Type 2 error of 20%, the estimated sample size was 100 patients per arm.

### Eligibility criteria

#### Inclusion criteria

Breast cancer patients over 18 years of age undergoing immediate or delayed breast reduction at Hospital das Clinicas Samuel Libânio will be considered eligible for participation.

#### Exclusion criteria

Patients with body mass index (BMI) above 35Kg/m^2^, those with usual contraindications for breast reconstruction procedures, such as diabetes or heavy smoking, will be excluded. Patients whose dressings get wet in the first 24 hours after operation, thus requiring their change, will also be excluded.

### Groups’ assignment, randomization and allocation concealment

Two hundred patients will be prospectively enrolled after giving informed consent. Patients will be randomly assigned to group I (n = 100), which will have dressings removed on the first postoperative day or to group II (n = 100), whose dressings will be removed on the sixth postoperative day. The rationale for comparing 24 hours versus 144 hours of dressing include CDC recommendation of protecting an incision with a sterile dressing for 24 to 48 hours [19] (group I) and the time of removing sutures (group II).

The allocation will be determined by a computer-generated sequence (Bioestat 5.0, Instituto de Desenvolvimento Sustentável Mamirauá, Belém, PA, Brazil). A numbered and sealed opaque envelope will be opened on the first postoperative day to reveal the group to which the patient is allocated.

### Baseline procedures and interventions

Patients will take a shower with liquid detergent-based chlorhexidine 4% prior to the operation [36], and an alcoholic solution of chlorhexidine 0.5% will be used for the antisepsis of the surgical site in the operating room [37]. Patients will undergo immediate or delayed breast reconstruction under general anesthesia, by the use of flaps and/or implants and, performed by the same surgical team. All patients will receive prophylactic antibiotics (cephazolin).

At the end of the operation, the surgical site will be cleansed with sterile physiological saline, a sample for quantitative skin culture will be obtained and a conventional gauze and tape dressing, which use is the established practice in our hospital, will be applied. Sutured wounds will be completely covered with four layers of dry sterile cotton gauze and fixed in place by a microporous adhesive tape. The surgical team will not be aware of the group to which the patient will be allocated.

Patients allocated to group I will be instructed to keep their wounds uncovered and to follow their usual personal hygiene routine, and patients in group II will be instructed not to wet the dressing.

Quantitative skin cultures will be obtained in the operating room immediately before applying the dressing, as well as immediately after the removal of dressing. In group I, an additional sample will be collected at the sixth postoperative day. A standard 5cm by 10 cm area (determined by a sterile pattern) over the surgical wound will be swabbed with a sterile cotton swab pre-moistened with sterile saline. This swab will be placed in a sterile container with 1.0 ml of saline and immediately conducted to the laboratory.

Patients will be discharged from the hospital on the first postoperative day after allocation, and they will return weekly for follow-up, for four weeks.

### Microbiological methods

Standard microbiological methods and criteria will be used to identify microorganisms [38]. Aliquots of 0.2 ml of the sample will be plated on hypertonic manitol (HM) agar, selective for staphylococci; on blood agar, to identify hemolytic colonies; on Sabouraud agar with chloramphenicol (0.05mg/ml), selective for fungi and yeasts; and on eosin-methylene blue (EMB) agar, selective for enterobacteria. The plates will be incubated aerobically for 48 hours at 37°C. The same laboratory technician will process all samples and, after 48 hours, the plates will be examined by a microbiologist.

Bacterial count results will be reported as colony forming units (CFU) per plate. Whenever CFU count in a plate exceeds 300, it will be scored as over 300. Staphylococci will be identified as coagulase-negative *Staphylococcus* sp. or *S. aureus* on the basis of Gram stain, the presence of hemolysis and on coagulase testing. The same microbiologist will assess all the plates. Both the laboratory technician and the microbiologist will be blinded.

### Assessment of postoperative infection

The Centers for Disease Control and Prevention (CDC) considers SSI to be an infection that occurs within 30 days after the operative procedure if no implant is left in place, or within one year if an implant is in place and the infection appears to be related to the operative procedure [39]. Thus, patients will be systematically followed-up once a week for 30 days regarding postoperative infection, by a single surgeon. Patients who receive an implant will have an additional assessment by the same surgeon, one year after the operation. The CDC definitions and classifications of SSI will be considered (Table 1) [39]. As with all CDC definitions of nosocomial infections, a surgeon’s diagnosis of infection will be considered an acceptable criterion for an SSI [39].

**Table 1:** **CDC definitions of SSI**[39]

**Superficial incisional SSI**	**Deep incisional SSI**	**Organ/Space SSI**
Involves only skin or subcutaneous tissue and meets at least one of the following:	Involves deep soft tissues (fascial and muscle layers) and meets at least one of the following:	Involves any part of the anatomy (organs or spaces) and meets at least one of the following:
• Purulent drainage from the superficial incision;	• Purulent drainage from the deep incision but not from the organ/space component of the surgical site;	• Purulent drainage from a drain that is placed through a stab wound into the organ/space;
• Organisms isolated from an aseptically obtained culture of fluid or tissue from the superficial incision;	• A deep incision that spontaneously dehisces or is deliberately opened by a surgeon when the patient has at least one of the following signs or symptoms: fever (>38°C), localized pain or tenderness, unless the incision is culture-negative;	• Organisms isolated from an aseptically obtained culture of fluid or tissue in the organ/space;
• At least one of the following signs or symptoms of infection: pain or tenderness, localized swelling, redness or heat, and the superficial incision is deliberately opened by surgeon unless the incision is culture-negative;	• An abscess or other evidence of infection involving the deep incision is found on direct examination, during reoperation, or by histopathologic or radiologic examination;	• An abscess or other evidence of infection involving the organ/space that is found on direct examination, during reoperation, or by histopathologic or radiologic examination;
• Diagnosis of superficial incisional SSI by the surgeon or attending physician.	• Diagnosis of deep incisional SSI by the surgeon or attending physician.	• Diagnosis of an organ/space SSI by the surgeon or attending physician.

### Patients’ assessments of dressing wear time

On their return in the second week after operation, patients will be asked to rate their dressing wear time with regard to safety, comfort and convenience, by the use of a 5-point rating scale (Table 2) [35].

**Table 2:** Patients’ self-assessment of dressing wear time

Please rate the **dressing wear time** following your breast surgery in regard to:				
**Safety**				
( ) Excellent	( ) Very good	( ) Good	( ) Fair	( ) Poor
**Comfort**				
( ) Excellent	( ) Very good	( ) Good	( ) Fair	( ) Poor
**Convenience**				
( ) Excellent	( ) Very good	( ) Good	( ) Fair	( ) Poor
Regardless of how many time your dressing was left in place, **if you had the choice**, would you prefer to keep the dressing for one day or for six days?				
( ) 1 day		( ) 6 days		

### Outcomes measures

The primary outcome is the incidence of SSI, which will be defined on the basis of CDC’s definitions [39]. Skin colonization rates and patients’ preferences regarding dressing wear time are secondary outcomes.

### End points

Participation is considered complete after the 30^th^ postoperative assessment day if no implants have been used, or after the 12^th^ postoperative month if an implant was used. Another exit point is if the dressings of group II patients get wet before the sixth postoperative day, thus requiring withdrawal from the study.

### Statistical analysis

The rejection level for the null hypothesis will be fixed at 5% (α ≤ 0.05). The Mann-Whitney test will be used to compare groups I and II with regard to age, BMI and duration of operation.

The Fisher test will be used to compare groups I and II regarding SSI occurrence. Logistic regressions will be applied to detect significant associations between variables such as age, BMI and duration of operation and the incidence of SSI.

For inter-group assessment of skin colonization, the Mann-Whitney test will be used to compare groups I and II pre-dressing and at the sixth postoperative day. These tests will be applied independently for each medium used. Because skin colonization could play a role in SSI rates, an intra-group assessment of skin colonization will also be performed. For this assessment, in group I Friedman two-way analysis of variance will be used to assess the differences in number of CFU among the three moments (pre-dressing, first and sixth postoperative day). Whenever the difference is significant, the Friedman two-way analysis of variance will be complemented by the multiple comparisons test to determine which moment significantly differed from the others. In group II, the Wilcoxon test will be used to compare the number of CFU pre-dressing and at the sixth postoperative day.

The Kolgomorov-Smirnov test will be applied to compare the two groups regarding patients’ assessments of safety, comfort and convenience. The Fisher test will be used to compare groups I and II regarding patients’ choice (one day or six days).

Statistical analysis will be performed using SPSS (Statistical Package for Social Sciences, Inc., Chicago, IL, USA) v.18 and Bioestat 5.0 (Instituto de Desenvolvimento Sustentável Mamirauá, Belém, PA, Brazil).

## Discussion

SSI is a major source of adverse events in patients undergoing breast cancer surgical procedures, generating psychological issues, increased duration of hospitalization and costs, as well as delay in commencing postoperative adjuvant therapies [2,5,11]. Consequently, surgeons should make a determined effort to prevent SSI. As protocols and guidelines should be founded on evidence-based medicine principles, well-designed studies are essential to support or to adjust clinical practice [20].

Wound management is a key area in preventing SSI [21]. However, few studies have focused on the management of the wound after closure as a method of reducing infection [34]. This randomized trial focuses on the influence of dressing wear time in SSI rates. Since the greater the degree of surgical wound contamination, the higher the risk for infection [2,19], skin colonization assessment is a secondary aim of this study.

The management of surgical wounds should involve the principle of minimizing harm, and patient preference and tolerance must also be considered [30]. Thus, assessment of patients’ perceptions of dressing wear time is another secondary aim in this trial. Some authors pointed out that omitting a dressing after the first 24 postoperative hours could be convenient for patients, allowing them to carry out their personal hygiene more easily [24]. However, other authors observed that dressings are comforting to patients by masking their scars without increasing SSI rates [23,26,35]. Considering the mutilating characteristics of breast cancer treatment, we hypothesize that patients, particularly those who undergo immediate breast reconstruction, might prefer to keep their dressings in place for a longer time, thus delaying the moment of seeing their reconstructed breasts.

CDC’s guidelines for managing surgical wounds that are closed primarily instruct patients to keep their wounds dry and covered for 24 to 48 hours [19], but the ideal dressing wear time following breast surgery remains an unsolved issue. The results of this trial may support standard recommendations regarding dressing wear time after breast reconstruction.

## Trial status

Recruitment is on-going (134 patients had been operated on by the end of December 2012).

## Abbreviations

CDC: Centers for Disease Control and Prevention;SSI: surgical site infection;BMI: body mass index;CFU: colony forming units;HM: hypertonic manitol;EMB: eosin-methylene blue

## Competing interests

The authors declare that they have no competing interests.

## Authors’ contributions

DFV conceived the study and participated in study design, surgical procedures, and manuscript draft. JVF and TBM were involved in surgical procedures and patients’ follow-up. CAVD and EMLS performed microbiology analysis and participated in interpretation of data. WEA contributed to acquisition of data. NFN participated in the analysis and interpretation of data. LMF participated in the design and coordination of the study. All authors contributed to the development of study protocol and all of them read and approved the final version of the manuscript.

## References

[B1] Gravante G Caruso R Araco A Cervelli V Infections after plastic procedures: incidences, etiologies, risk factors, and antibiotic prophylaxis Aeth Plast Surg 2008 32 243 251 10.1007/s00266-007-9068-8 18080159

[B2] Anderson DJ Kaye KS Staphylococcal surgical site infections Infect Dis Clin N Am 2009 23 53 72 10.1016/j.idc.2008.10.004 19135916

[B3] Nichols RL Preventing surgical site infections: a surgeon’s perspective Emerg Infect Dis 2001 7 220 224 10.3201/eid0702.010214 11294711 PMC2631713

[B4] Andenaes K Amland PF Lingaas E Abyholm F Samdal F Giercksky KE A prospective, randomized surveillance study of postoperative wound infections after plastic surgery: a study of incidence and surveillance methods Plast Reconstr Surg 1995 96 948 956 10.1097/00006534-199509001-00028 7652070

[B5] Olsen MA Chu-Ongsakul S Brandt KE Dietz JR Mayfield J Fraser V Hospital-associated costs due to surgical site infection after breast surgery Arch Surg 2008 143 53 60 10.1001/archsurg.2007.11 18209153

[B6] Alvarez JM Use of an occlusive dressing for two weeks reduces the incidence of sternal wound infections ANZ J Surg 2005 75 179 180 10.1111/j.1445-2197.2005.03320.x 15777404

[B7] Olsen MA Lefta M Dietz JR Brandt KE Aft R Matthews R Mayfield J Frasere VJ Risk factors for surgical site infection after major breast operation J Am Coll Surg 2008 207 326 335 10.1016/j.jamcollsurg.2008.04.021 18722936 PMC3618680

[B8] Perotti JA Castor SA Perez PC Zins JE Antibiotic use in aesthetic surgery: a national survey and literature review Plast Reconstr Surg 2002 109 1685 1693 10.1097/00006534-200204150-00033 11932619

[B9] Tejirian T DiFronzo LA Haigh PI Antibiotic prophylaxis for preventing wound infection after breast surgery: a systematic review and meta-analysis J Am Coll Surg 2006 203 729 734 10.1016/j.jamcollsurg.2006.07.013 17084336

[B10] Hall JC Willsher PC Hall JL Randomized clinical trial of single-dose antibiotic prophylaxis for non-reconstructive breast surgery Br J Surg 2006 93 1342 1346 10.1002/bjs.5505 16989011

[B11] Xue DQ Qian C Yang L Wang XF Risk factors for surgical site infections after breast surgery: a systematic review and meta-analysis Eur J Surg Oncol 2012 38 375 381 10.1016/j.ejso.2012.02.179 22421530

[B12] Tran CL Langer S Broderick-Villa G DiFronzo LA Does reoperation predispose to postoperative wound infection on women undergoing operation for breast cancer? Am Surg 2003 69 852 856 14570362

[B13] Chen J Gutkin Z Bawnik J Postoperative infections in breast surgery J Hosp Infect 1991 17 61 65 10.1016/0195-6701(91)90078-M 1672325

[B14] Bertin ML Crowe J Gordon SM Determinants of surgical site infection after breast surgery Am J Infect Control 1998 26 61 65 10.1016/S0196-6553(98)70062-8 9503114

[B15] Alderman AK Wilkins EG Kim HM Lowery JC Complications in postmastectomy breast reconstruction: two-year results of the Michigan Breast Reconstruction Outcome Study Plast Reconstr Surg 2002 109 2265 2274 10.1097/00006534-200206000-00015 12045548

[B16] Nahabedian MY Tsangaris T Momen B Manson PN Infectious complications following breast reconstruction with expanders and implants Plast Reconstr Surg 2003 112 467 476 10.1097/01.PRS.0000070727.02992.54 12900604

[B17] Sørensen LT Hørby J Friis E Pilsgaard B Jørgensen T Smoking as a risk factor for wound healing and infection in breast cancer surgery ESJO 2002 28 815 820 10.1053/ejso.2002.1308 12477471

[B18] Landes G Harris PG Lemaine V Perreault I Sampalis JS Brutus JP Lessard L Dionyssopoulos A Nikolis A Prevention of surgical site infection and appropriateness of antibiotic prescribing habits in plastic surgery J Plast Recontr Aesth Surg 2008 61 1347 1356 10.1016/j.bjps.2008.02.008 18558522

[B19] Mangram AJ Horan TC Pearson ML Silver LC Jarvis WR The Hospital Infection Control Practices Advisory Committee Guideline for prevention of surgical site infection Am J Infect Control 1999 27 97 134 10.1016/S0196-6553(99)70088-X 10196487

[B20] Segers P de Jong AP Spanjaard L Ubbink DT de Mol BAJM Randomized clinical trial comparing two options for postoperative incisional care to prevent poststernotomy surgical site infections Wound Rep Reg 2007 15 192 196 10.1111/j.1524-475X.2007.00204.x 17352750

[B21] Sticha RS Swiriduk D Wertheimer SJ Prospective analysis of postoperative wound infections using an early exposure method of wound care J Foot Ankle Surg 1998 37 286 291 10.1016/S1067-2516(98)80064-1 9710780

[B22] Heal C Buettner P Raasch B Browning S Graham D Bidgood R Campbell M Cruikshank R Can sutures get wet? Prospective randomized controlled trial of wound management in general practice BMJ 2006 332 1053 1056 10.1136/bmj.38800.628704.AE 16636023 PMC1458594

[B23] Cho CY Lo JS Dressing the part Dermatol Clin 1998 16 25 47 10.1016/S0733-8635(05)70485-X 9460576

[B24] Chrintz H Cordtz TO Harreby JS Waaddegaard P Larsen SO Need for surgical wound dressing Br J Surg 1989 76 204 205 10.1002/bjs.1800760232 2702460

[B25] Lionelli GT Lawrence WT Wound dressings Surg Clin N Am 2003 83 617 638 10.1016/S0039-6109(02)00192-5 12822729

[B26] Wynne R Botti M Stedman H Holsworth L Harinos M Flavell O Manterfield C Effect of three wound dressings on infection, healing comfort, and cost in patients with sternotomy wounds: a randomized trial Chest 2004 125 43 49 10.1378/chest.125.1.43 14718419

[B27] Michie DD Hugill JV Influence of occlusive and impregnated gauze dressings on incisional healing: a prospective, randomized, controlled study Ann Plast Surg 1994 32 57 64 10.1097/00000637-199401000-00011 8141537

[B28] Benito P De Juan A Cano M Elena E An easy and comfortable way of maintaining dressings in breast surgery Plast Reconstr Surg 2008 121 680 681 10.1097/01.prs.0000298525.63082.74 18300997

[B29] Al-Benna S An easy and comfortable way of maintaining dressings in breast surgery - reply Plast Reconstr Surg 2008 121 680 681 10.1097/01.prs.0000298525.63082.74 18300997

[B30] Paddle-Ledinek JE Nasa Z Cleland H Effect of different wound dressings on cell viability and proliferation Plast Reconstr Surg 2006 117 Suppl 110S 118S 10.1097/01.prs.0000225439.39352.ce 16799377

[B31] Holm C Petersen JS Gronboek F Gottrup F Effects of occlusive and conventional gauze dressings on incisional healing after abdominal operations Eur J Surg 1998 164 179 183 9562277 10.1080/110241598750004616

[B32] Jones VJ The use of gauze: will it ever change? Int Wound J 2006 3 79 86 10.1111/j.1742-4801.2006.00215.x 17007339 PMC7951218

[B33] Thomas DW Hill CM Lewis MAO Stephens P Walker R Von Der Weth A Randomized clinical trial of the effect of semi-occlusive dressings on the microflora and clinical outcome of acute facial wounds Wound Rep Reg 2000 8 258 263 10.1046/j.1524-475x.2000.00258.x 11013016

[B34] Rosenfeldt FL Negri J Holdaway D Davis BB Mack J Grigg MJ Miles C Esmore DS Occlusive wrap dressing reduces infection rate in saphenous vein harvest site Ann Thorac Surg 2003 75 101 105 10.1016/S0003-4975(02)04121-8 12537200

[B35] Veiga-Filho J Veiga DF Sabino-Neto M Damasceno CAV Sales EM Garcia ES Oliveira IB De Simoni LF Juliano Y Ferreira LM Dressing wear time after reduction mammaplasty: a randomized controlled trial Plast Reconstr Surg 2012 129 1e 7e 10.1097/PRS.0b013e3182361ee9 22186564

[B36] Veiga DF Damasceno CAV Veiga-Filho J Figueiras RG Vieira RB Garcia ES Silva VV Novo NF Ferreira LM Randomized controlled trial on the effectiveness of chlorhexidine showers before elective plastic surgical procedures Infect Control Hosp Epidemiol 2009 30 77 79 10.1086/592980 19046051

[B37] Veiga DF Damasceno CAV Veiga-Filho J Figueiras RG Vieira RB Florenzano FH Juliano Y Ferreira LM Povidone-iodine (PVP-I) versus chlorhexidine in antisepsis before elective plastic surgery procedures: randomized controlled trial Plast Reconstr Surg 2008 122 170e 171e 10.1097/PRS.0b013e318186cd7f 18971711

[B38] Trabulsi LR Alterthum F Microbiologia 2005 4 São Paulo: Atheneu

[B39] Horan TC Gaynes RPG Martone WJ Jarvis WR Emori TG CDC definitions of nosocomial surgical site infections, 1992: a modification of CDC definitions of surgical wound infections Infect Control Hosp Epidemiol 1992 13 606 608 10.1086/646436 1334988

